# Adding behavior traits to select for heat tolerance in dairy cattle

**DOI:** 10.3168/jdsc.2023-0421

**Published:** 2024-01-15

**Authors:** P. Lemal, M-N. Tran, H. Atashi, M. Schroyen, N. Gengler

**Affiliations:** 1 Gembloux Agro-Bio Tech, University of Liège, 5030 Gembloux, Belgium; 2 Elevéo, Association Wallonne des Eleveurs, 5590 Ciney, Belgium; 3 Department of Animal Science, Shiraz University, 71441-13131 Shiraz, Iran

## Abstract

The increase in periods of heat waves leads to an increase in heat stress events in dairy cattle leading to welfare issues, production losses, and health issues. However, the low frequency of milk recording data makes genetic evaluation for heat tolerance still a challenge. A possible solution could be to add behavior data captured through sensors which are recorded permanently, mostly reported on a daily basis. The objective of this study was to evaluate the potential gain of adding behavior traits as proxies for genetic evaluation of heat tolerance. Behavior traits including activity time (ACT), rumination time (RUM), and eating time (EAT) were recorded for 453 Holstein cows equipped with SenseHub (Allflex Livestock Intelligence) collars from October 2019 to July 2022 in 6 herd located in the Walloon Region of Belgium. A multitrait reaction norm model based on separate temperature and humidity index (THI) thresholds was used. Results showed that behavior traits present not only interesting characteristics for genetic evaluation of heat tolerance but also for heat stress detection in farms. Indeed, sensors allow recording of behavior for all events of heat stress in lactating and nonlactating animals. Moderate heritability values were also found for the behavior traits (0.14 for ACT, 0.19 for RUM, and 0.12 for EAT), and a high ratio between the general and thermotolerance additive genetic variances was obtained. In addition, positive correlations of thermotolerance for ACT and EAT with thermotolerance for milk production (fat- and protein-corrected milk: 0.45 and 0.28, respectively) and negative genetic correlations of thermotolerance for ACT with somatic cells (somatic cell score: −0.39) were estimated. The genetic correlation matrix allows us to explain a high part of the variation for the reaction to heat stress of 2 economic traits (fat- and protein-corrected milk: 59% and somatic cell score: 31%) based on behavior data. Based on these results, behavior traits could be used to assess heat stress in nonlactating cattle for which the number of genetic evaluations for heat tolerance is still limited.

## Highlights

• Behavior traits could provide relevant data to be used in genetic evaluation systems for heat tolerance in dairy cattle.
• Behavior traits could extend records for heat stress to nonlactating animals.
• Activity time seems to be the more promising behavioral trait for heat stress detection and genetic evaluation for heat tolerance.

## Graphical Abstract

**Figure:**
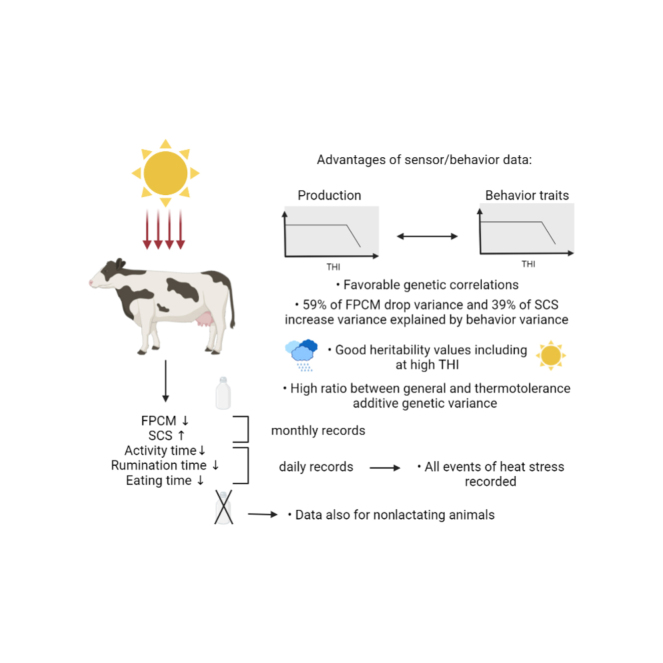
**Summary** The objective of this study was to evaluate the gain of adding sensor/behavior data to the genetic evaluation systems for heat tolerance in dairy cattle. Several important results were found as favorable genetic correlations for thermotolerance among fat- and protein-corrected milk (FPCM), somatic cell score (SCS), and behavior traits; ability of behavior data to predict drop of FPCM during heat stress; high ratio between the general additive genetic variance and the thermotolerance additive genetic variance; and good heritability values obtained for behavior data including at the maximum temperature-humidity index (THI). Moreover, the daily availability of sensor data allows for the recording of all heat stress events, and the possibility of obtaining records on nonlactating animals opens new opportunities for genetic evaluation systems for heat tolerance.

Heat stress is well known to negatively affect dairy cattle production, welfare, and health (Becker et al., 2020). Indeed, dairy cows are especially sensitive to heat due to their high metabolic rate (Wang et al., 2020). In the United States, it has been predicted that temperature elevation by 2050 could lead to a decrease of 1.4 kg/d per cow of milk production resulting in an economic loss of $1.7 billion per year (Mauger et al., 2015; Wankar et al., 2021). A proposed solution to alleviate heat stress in a permanent and cumulative way is through genetic selection for thermotolerant animals (Garner et al., 2016; Osei-Amponsah et al., 2019). The most direct way to measure heat stress is to use body temperature as phenotype, but routine measurements are especially difficult to be established in a large-scale system (Ji et al., 2020). Conversely, production performances are available at a large scale through milk recording and are thus frequently used to evaluate heat tolerance in dairy cattle (Hammami et al., 2015). However, milk recording intervals are counted in weeks (ICAR, 2022), which drastically limits the number of records available during hot days (Carabaño et al., 2019). An alternative could be to combine milk recording and behavior data collected using sensors. The objective of this work was thus to evaluate the potential value of adding behavior data for genetic evaluation of heat tolerance in dairy cows.

Behavior data were obtained for 453 Holstein cows distributed in 6 herds equipped with SenseHub (Allflex Livestock Intelligence) collars from October 2019 to July 2022 in the Walloon Region of Belgium. The used sensors provided daily information for activity time (**ACT**), rumination time (**RUM**), and eating time (**EAT**). Milk recording information including milk yield, fat percentage, protein percentage, and SCC were obtained for 1,740 Holstein cows (which included the 453 cows) from the same 6 herds from 2015 to 2022 to fit with the meteorological data obtained during the same period. Meteorological data included hourly temperature (T; °C) and hourly relative humidity (**RH**; %) from the nearest weather station of every herd. The hourly temperature-humidity index (**THI**) was calculated following this formula (NRC, 1971):

[1] THI = [(1.8 × T) + 32] – {(0.55 – 0.0055 × RH) × [(1.8 × T) – 26]}.

The daily THI was then defined as the mean of the hourly THI of the concerned day. To consider the delay between the onset of high THI and the cow reaction to heat stress, the mean THI of the day and the 3 previous days was then used. Indeed, our preliminary investigation showed that this time point presented the highest variability with THI.

To prevent the dilution effect of a decrease in milk yield on fat and protein contents, fat- and protein-corrected milk (**FPCM**) was used instead of milk yield, fat percentage, and protein percentage separately. The FPCM was calculated as follows (FAO, 2010):

[2] FPCM = milk yield × (0.337 + 0.116 × fat percentage + 0.06 × protein percentage).

The SCC data were converted to SCS using the following formula (Wiggans and Shook, 1987):

[3] SCS = [log_2_ (SCC/100,000)] + 3 with minimum SCS = 0.1.

For the 5 traits (FPCM, SCS, ACT, RUM, and EAT), data outside the range mean ± 3 standard deviations (**SD**) were excluded. Descriptive statistics are presented in Table 1.

**Table 1:** Descriptive statistics for the studied traits and the temperature-humidity index (THI)

Trait^1^ (unit)	Mean	Minimum	Maximum	SD	N
FPCM (kg)	27.36	1.48	59.95	8.02	32,154
SCS	2.30	0.10	7.78	1.66	31,564
ACT (min/24 h)	294.23	141.25	449.73	39.01	130,867
RUM (min/24 h)	580.62	328.00	804.00	68.24	130,848
EAT (min/24 h)	297.01	49.50	545.00	80.05	131,907
THI	49.85	23.94	73.77	9.62	161,949

1 FPCM = fat- and protein-corrected milk; ACT = activity time; RUM = rumination time; EAT = eating time.

The THI thresholds at which we considered that heat stress starts to affect the different traits were estimated following a multitrait model based on the model proposed by McWhorter et al. (2022):

[4] *y_ijklmnop_* = THI*_ij_* + HY*_ik_* + lact*_il_* + (DIM × s)*_im_* + age*_in_* + a*_io_* + pe*_io_* + *e_ijklmnop_*,

where *y_ijklmnop_* is the analyzed trait *i* (FPCM, SCS, ACT, RUM, or EAT), THI*_ij_* is the categorical fixed effect for the mean THI of the day and the 3 previous days of class *j*, HY*_ik_* is the categorical fixed effect for the herd-year of class *k*, lact*_il_* is the categorical fixed effect for the lactation number of class *l* (lactation 1, 2, 3, 4, and 5+), (DIM × s)*_im_* is the categorical fixed effect for the combination of the classes of DIM (classes of 30 d of DIM) and the season of calving of class *m*, age*_in_* is the categorical fixed effect for the age at calving of class *n* (5 classes), a*_io_* is the additive genetic random effect for animal *o*, pe*_io_* is the permanent environmental random effect for animal *o*, and *e_ijklmnop_* is the residual.

The results for the THI effect and the relative THI effect (THI effect obtained in Equation 4/phenotypic SD) were then represented as a function of the THI to set the THI threshold for every trait. Polynomials of degree 5 were added to help to determine the thresholds (Figure 1). The results show a decrease of the 3 behavior traits with increasing THI. In the literature, Ramón-Moragues et al. (2021) obtained a decrease of RUM and EAT but also an increase of ACT for heat-stressed cows compared with non-heat-stressed cows. Similarly, Abeni and Galli (2017) showed a lower RUM and a slightly higher ACT during a hot day (THI around 80) compared with a cooler day (THI around 70). Conversely, Hut et al. (2022) showed a decrease of walking time, a slight increase of RUM with increasing THI, but also a decrease of EAT. On this basis, the reduction of EAT during heat stress seems to be accepted, whereas the effect of ACT varies a lot with studies. This could be due to various factors including the devices used, the population studied, and the intensity and duration of the heat period.

**Figure 1:**
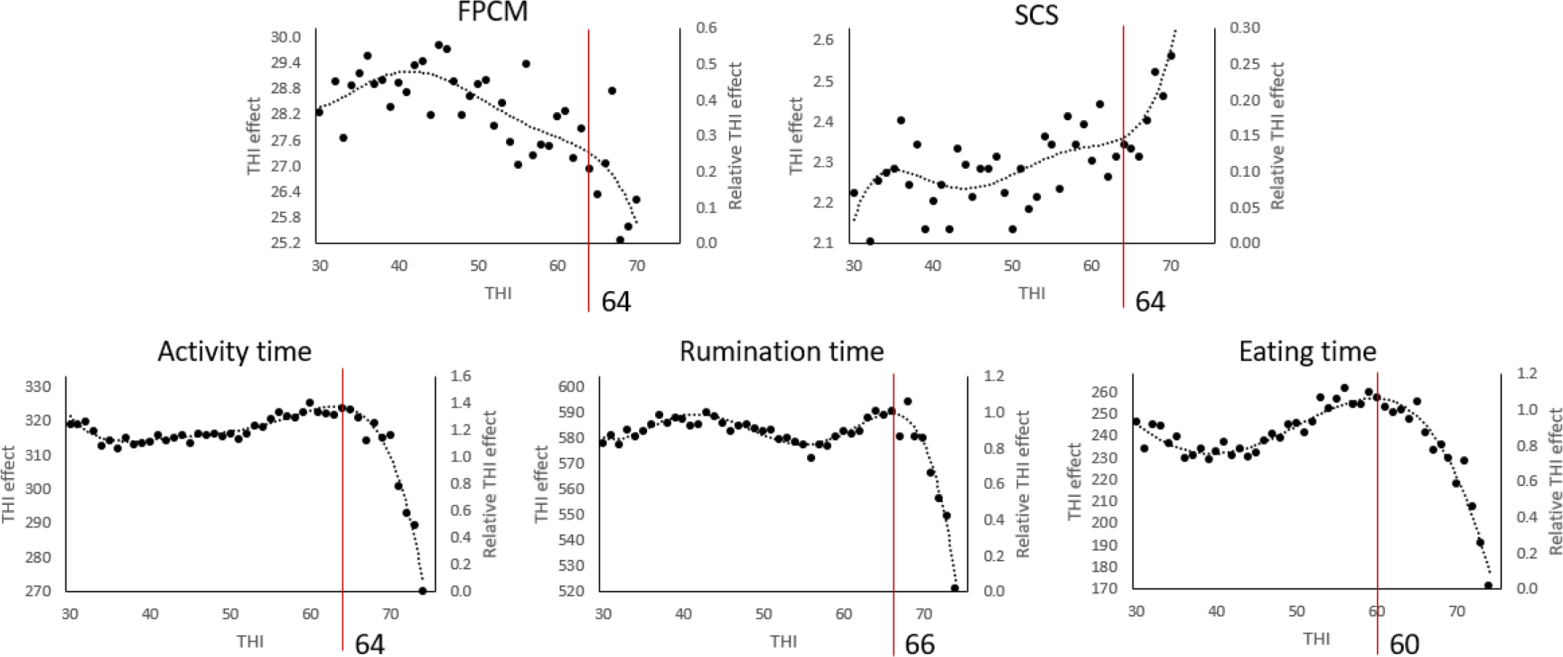
Temperature-humidity index (THI) effect estimated with Equation 4 and represented as THI effect and relative THI effect (THI effect/phenotypic SD) for the 5 traits studied. Dotted line: polynomials of degree 5. Red lines: THI thresholds. FPCM = fat- and protein-corrected milk.

We chose THI thresholds of 60 for EAT; 64 for FPCM, SCS, and ACT; and 66 for RUM (Figure 1). All thresholds appear at similar THI, but behavior data thresholds were clearer than FPCM and SCS thresholds. This could be due to the higher frequency of behavior data. By looking at the relative THI effect, it seems also higher for behavior data but the maximum THI for the behavior data (74) was higher than for FPCM and SCS (70). In this way, a bigger reaction should probably be visible at higher THI also for FPCM and SCS.

Based on the thresholds, a multitrait reaction norm model was then performed based on the model proposed by McWhorter et al. (2022):

[5] *y_ihlmnop_* = HTD*_ih_* + lact*_il_* + (DIM × s)*_im_* + age*_in_* + a*_io_* + *α_io_*[*f*(THI)] + pe*_io_* + *π_io_*[*f*(THI)] + *e_ihlmnop_*,

where *y_ihlmnop_* is the analyzed trait *i* (FPCM, SCS, ACT, RUM, or EAT), HTD*_ih_* is the categorical fixed effect for the herd test-day of class *h*, *α_io_* is the slope of the regression on the THI for the random additive genetic effect (thermotolerance additive genetic effect) for animal *o*, *π_io_* is the slope of the regression on the THI for the random permanent environmental effect (thermotolerance permanent environment effect) for animal *o*, *f*(THI) = 0 if THI_test-day_ < THI_threshold_ and *f*(THI) = THI_test-day_ − THI_threshold_ if THI_test-day_ ≥ THI_threshold_, and *e_ihlmnop_* is the residual.

All data were prepared in the SAS environment (SAS version 9.4, SAS Institute Inc., Cary, NC). The (co)variance components estimation and their standard errors (**SE**) were estimated with BLUPF90+ programs from the BLUPF90 family of programs (Misztal et al., 2014).

Based on the estimated (co)variances, genetic correlations for thermotolerance for every trait were estimated and listed in Table 2.

**Table 2:** Genetic correlations (± SE) for thermotolerance, heritability values at the temperature-humidity index (THI) of the thresholds or at the maximum THI (THI_max_), and the ratio between the general additive genetic variance $\left(\sigma_{a}^{2}\right)$ and the thermotolerance additive genetic variance $\left(\sigma_{\alpha }^{2}\right)$^1^

Trait	Genetic correlation for thermotolerance ± SE				Heritability ± SE		σα2/σα2σa2σa2 ± SE
	FPCM	SCS	ACT	RUM	THI_threshold_	THI_max_	
FPCM					0.15 ± 0.02	0.08 ± 0.18	0.006 ± 0.015
SCS	−0.40 ± 0.18				0.08 ± 0.02	0.09 ± 0.12	0.010 ± 0.044
ACT	0.45 ± 0.62	−0.39 ± 0.18			0.14 ± 0.06	0.31 ± 0.25	0.023 ± 0.093
RUM	−0.02 ± 0.46	−0.10 ± 0.43	0.73 ± 0.66		0.19 ± 0.05	0.17 ± 0.29	0.053 ± 0.021
EAT	0.28 ± 0.70	0.06 ± 0.55	−0.01 ± 0.18	−0.12 ± 0.36	0.12 ± 0.05	0.09 ± 0.21	0.005 ± 0.028

1 FPCM = fat- and protein-corrected milk; ACT: activity time; RUM: rumination time; EAT: eating time.

Genetic correlations for thermotolerance between traits *i* and *j* were estimated using the Pearson correlation formula:

[6] rα(i,j)=(covα(i,j))/(covα(i,j))[√(σαi2σαj2)][√(σαi2σαj2)],

where *r_α_*_(_*_i,j_*_)_ is the correlation for the thermotolerance additive genetic effect between trait *i* and trait *j*, cov*_α_*_(_*_i_*_,_*_j_*_)_ is the covariance for the thermotolerance additive genetic effect between trait *i* and trait *j*, $\sigma_{\alpha i}^{2}$ is the variance for the thermotolerance additive genetic effect of trait *i*, and $\sigma_{\alpha j}^{2}$ is the variance for the thermotolerance additive genetic effect of trait *j*.

The results show a negative genetic correlation for thermotolerance between FPCM and SCS (−0.40) suggesting that by selecting cows with a lower decrease of FPCM during heat stress events, we are also indirectly selecting cows with a lower increase of SCS during heat stress. This was consistent with the general negative genetic correlation observed between milk yield and SCS. Indeed, animals with higher SCS have the tendency to produce less milk (Banos and Shook, 1990). Similarly, by looking at the genetic correlations between behavior data and FPCM (0.45 for ACT and 0.28 for EAT), we can expect that by selecting cows for a lower decrease of ACT and EAT during events of heat stress we also keep cows with a lower decrease of production during heat stress. Likewise, by selecting cows with a lower drop of ACT during hot days we also indirectly would tend to select cows with a lower increase of SCS during these days. We also looked at eigenvalues and the associated eigenvectors. The first eigenvector of the genetic correlation matrix for thermotolerance for the 5 traits represented 42% of the variability and the standardized coefficients were 0.42, −0.43, 0.65, 0.47, and 0.03, respectively, for FPCM, SCS, ACT, RUM, and EAT. As expected, the same direction of variation was observed for behavior data and FPCM and an opposite direction of variation was found for behavior data and SCS except for EAT, which was neutral.

Regarding these results, behavior information could be used to predict, at least partially, economic traits like FPCM and SCS thermotolerance. To confirm this hypothesis, we estimated the proportion of the variance for thermotolerance for FPCM and SCS that could be explained by behavior data using the following formula:

[7] p*_i_* = b[R*_α_*_(ACT-RUM-EAT)_]b′, where b = [r*_α_*_(_*_i_*_,ACT-RUM-EAT)_]′ [R*_α_*_(ACT-RUM-EAT)_]^−1^,

where R*_α_*_(ACT-RUM-EAT)_ is the 3 × 3 additive genetic correlation matrix between the 3 behavior traits (ACT, RUM, and EAT) for the thermotolerance effects and r*_α_*_(_*_i_*_,ACT-RUM-EAT)_ is the 3 × 1 correlation vector for the thermotolerance additive genetic effect between the performance trait *i* and the 3 behavior traits.

To also consider the general additive genetic effect of the behavior traits, the formula was adapted:

[8] p*_i_* = b[R*_αa_*_(ACT-RUM-EAT)_]b′, where b = [r*_αa_*_(_*_i_*_,ACT-RUM-EAT)_]′ [R*_αa_*_(ACT-RUM-EAT)_]^−1^,

where R*_αa_*_(ACT-RUM-EAT)_ is the 6 × 6 correlation matrix for the thermotolerance additive genetic effect and the general additive genetic effect between the 3 behavior traits and r*_αa_*_(_*_i_*_,ACT-RUM-EAT)_ is the 6 × 1 correlation vector between the performance trait *i* for the thermotolerance additive genetic effect and the 3 behavior traits for the thermotolerance additive genetic effect and the general additive genetic effect.

By using the thermotolerance information only as described in Equation 7, behavior traits explained 51% of the variance of thermotolerance for FPCM and 23% for SCS. By adding the general additive variance for the behavior traits as described in Equation 8, the percentage of variability explained increased to 59% for FPCM and to 31% for SCS.

Based on variance estimations, heritability values for every trait (Table 2) were also calculated following Ravagnolo and Misztal (2000):

[9] $$\begin{array}{c} h_{f\left(THI\right)}^{2}=[\sigma_{a}^{2}+f{\left(THI\right)}^{2}\sigma_{\alpha }^{2}+2f\left(THI\right)\sigma_{a\alpha }]/[\sigma_{a}^{2}+f{\left(THI\right)}^{2}\sigma_{\alpha }^{2}+2f\left(THI\right)\sigma_{a\alpha }+\sigma_{pe}^{2}\\ +f{\left(THI\right)}^{2}\sigma_{\pi }^{2}+2f\left(THI\right)\sigma_{pe\pi }+\sigma_{e}^{2}], \end{array}$$

where $[h_{f\left(THI\right)}^{2}$ is the heritability at a given THI with *f*(THI) = 0 if THI < THI_threshold_ and *f*(THI) = THI − THI_threshold_ if THI ≥ THI_threshold_, $\sigma_{a}^{2}$ is the variance for the general additive genetic effect, $\sigma_{\alpha }^{2}$ is the variance for the thermotolerance additive genetic effect, *σ_aα_* is the covariance between the general and the thermotolerance additive genetic effects, $\sigma_{pe}^{2}$ is the variance for the general permanent environmental effect, $\sigma_{\pi }^{2}$ is the variance for the thermotolerance permanent environmental effect, *σ_peπ_* is the covariance between the general and the thermotolerance permanent environmental effects, and $\sigma_{e}^{2}$ is the residual variance.

Heritability values were relatively similar at the THI of the thresholds and at the maximum THI except for ACT. This could be due to the low maximum THI observed in Belgium compared with hotter countries. The heritability of SCS was in line with the literature for Holstein cows (Kheirabadi and Razmkabir, 2016; Tiezzi et al., 2020), whereas heritability for FPCM was relatively low (Manzanilla Pech et al., 2014; Lassen and Løvendahl, 2016). Concerning sensor data, not so much information was available because they are still rarely used in genetics studies. For activity measurement with different devices, heritability values vary from 0.10 to 0.45 (Schöpke, 2014; Poppe et al., 2022), which included the value of 0.19 obtained in this study. For RUM, Moretti et al. (2018) obtained higher values (0.31 to 0.36) with similar sensors. Finally, for EAT, Cavani et al. (2022) estimated a heritability of 0.23 for the average daily time at the feeder. In general, the heritability values obtained in this study were lower compared with the literature. This could be due to differences among populations or the low number of cows used in our study.

The values for the ratio between general additive genetic variance $\left(\sigma_{a}^{2}\right)$ and thermotolerance additive genetic variance $\left(\sigma_{\alpha }^{2}\right)$ were also estimated (Table 2). The results showed a higher ratio for ACT and RUM compared with FPCM and SCS suggesting that a higher part of the genetic variance was associated with thermotolerance for these 2 traits.

Based on the results, ACT seems to be the most interesting behavior trait. Indeed, it presents the most interesting genetic correlations for thermotolerance with FPCM (0.45) and SCS (−0.39), a high ratio between additive genetic variances (0.023) and the highest heritability at the maximum THI (0.31). The principal component analysis showed the same direction of variation for thermotolerance between ACT and FPCM and an opposite direction of variation between ACT and SCS. In addition, the interest of using ACT to evaluate heat tolerance was supported by Poppe et al. (2022) who showed that animals with a lower activity drop (step count in their case) during disturbances could be more resilient in general.

Based on the results discussed, behavior data present several qualities for genetic evaluation for heat tolerance: (1) positive or neutral genetic correlations for thermotolerance with FPCM and negative or neutral with SCS allowing indirect selection for thermotolerance of FPCM and SCS by selecting for thermotolerance of behavior traits, (2) high ratio between the thermotolerance additive genetic effect and the general additive genetic effect, (3) heritability values allowing selection for behavior traits including at the maximum THI, (4) higher frequency of recording (daily) allowing the coverage of all events of heat stress and a better estimation in less time as shown by the smoother curves obtained for the THI effect in Figure 1. In addition, behavior traits explained 59% of the FPCM reaction to THI. In this way, behavior traits that are not directly economic traits would allow prediction of reactions of economic traits to heat. Finally, heat stress genetic evaluation based on behavior traits will be extendable to nonlactating animals like heifers, bulls, and beef cattle. In this way, in addition to being used for heat stress genetic evaluation for dairy cows, sensors could help to extend heat stress genetic evaluation systems for heat tolerance to all cattle. Indeed, we could expect that nonlactating animals with a high modification of behavior traits during heat stress events will also have high reduction of performances during heat in their subsequent lactations for heifers and dry cows, generate offspring more susceptible to heat for bulls, and present reduced growth performances due to heat for beef cattle. Currently, it is known that heat stress during the dry period has a negative effect on the production for the subsequent lactations (Fabris et al., 2019), but no studies to our knowledge highlighted that dry cows more susceptible to heat will become lactating cows showing a high drop of production during heat waves. Further studies are thus required to test these hypotheses.

The biggest current restraint of large-scale use of sensors and thus the availability of data for genetic evaluation is their cost. However, the first function of sensors is reproductive heat detection. Based on our results, genetic evaluation for heat tolerance should offer an additional purpose for sensor use. In addition, heat stress could also directly affect pregnancy rate by decreasing activity during hot periods. Indeed, the negative impact of heat stress on estrus expression including a lower increase of activity during reproductive heat could reduce the number of detected estrus and thus indirectly reduce the pregnancy rate (Schüller et al., 2017; Hansen, 2019). On this basis, cows with a high drop of activity during heat stress could also present a reduced pregnancy rate during this period.

Finally, behavior data could also be used for heat stress detection due to their daily recording pattern, clear THI thresholds, and high variation along the THI scale as shown by the relative THI effect we reported in this study.
